# In the Tumor Microenvironment, ETS1 Is an Oncogenic Immune Protein: An Integrative Pancancer Analysis

**DOI:** 10.1155/2022/7730433

**Published:** 2022-04-15

**Authors:** Zixuan Wu, Yanhui Jiang, Xuyan Huang, Minjie Cai, Kai Yuan, Peidong Huang, Zuhong Wang

**Affiliations:** ^1^Guangzhou University of Chinese Medicine, Guangzhou 510006, Guangdong Province, China; ^2^Shantou Health School, Shantou 515061, Guangdong Province, China; ^3^Yunnan University of Chinese Medicine, Kunming 650500, Yunnan Province, China; ^4^Kunming Municipal Hospital of Traditional Chinese Medicine, Kunming 650011, Yunnan Province, China

## Abstract

**Background:**

Previous research suggested that ETS1 (ETS proto-oncogene 1, transcription factor) could be useful for cancer immunotherapy. The processes underlying its therapeutic potential, on the other hand, have yet to be thoroughly investigated. The purpose of this study was to look into the relationship between ETS1 expression and immunity.

**Methods:**

TCGA and GEO provide raw data on 33 different cancers as well as GSE67501, GSE78220, and IMvigor210. In addition, we looked at ETS1's genetic changes, expression patterns, and survival studies. The linkages between ETS1 and TME, as well as its association with immunological processes/elements and the major histocompatibility complex, were explored to effectively understand the role of ETS1 in cancer immunotherapy. Three distinct immunotherapeutic cohorts were employed to examine the relationship between ETS1 and immunotherapeutic response.

**Results:**

ETS1 expression was shown to be high in tumor tissue. ETS1 overexpression is linked to a worse clinical outcome in individuals with overall survival. Immune cell infiltration, immunological modulators, and immunotherapeutic signs are all linked to ETS1. Overexpression of ETS1 is linked to immune-related pathways. However, no statistically significant link was found between ETS1 and immunotherapeutic response.

**Conclusions:**

ETS1 may be a reliable biomarker for tumor prognosis and a viable prospective therapeutic target for human cancer immunotherapy (e.g., KIRP, MESO, BLCA, KIRC, and THYM).

## 1. Introduction

ETS1 (ETS proto-oncogene 1, transcription factor) is a transcription factor that is mainly expressed in lymphoid cells. It is also an oncogene that is commonly elevated in human malignancies from various tissue origins [1, 2]. Nowadays, ETS1 is becoming increasingly popular as a possible biomarker and essential mediator in various cancers [3–5]. Several investigations have found that ETS1 can hinder cell differentiation in a variety of circumstances and increase its cancer-promoting activity by keeping cells immature and proliferating. As a result, ETS1 may help convert drug prospects into therapeutic anticancer strategies [6]. The link between ETS1 function and carcinogenesis, on the other hand, is still uncertain, which might be a hot study topic.

The world is today confronted with a significant public health issue: cancer incidence and mortality remain high. *Cancer* is a problematic sickness because tumors interact with the immune system [7, 8]. The tumor microenvironment (TME) is made up of many cells and plays an important role in the development, metastasis, and treatment resistance of human cancers [9, 10]. However, the method through which TME interacts with immune cells is unknown. TME has lately emerged as a new immunotherapy hotspot. Since immunotherapy with immune checkpoint blockade and other techniques, several therapeutic target-blocking medicines have been employed for cancer treatment [11]. As a result, immunophenotypes and the validation of new immune-related tumor treatment targets are crucial. However, research on ETS1's role in generalized cancer is sparse.

This researcher was interested in critical immune modulators and dynamic immunological markers such as tumor mutational burden (TMB) and microsatellite instability (MSI). Furthermore, the relationship between ETS1 expression and immune checkpoint inhibitors was investigated. Taking all of these factors into account, ETS1 was discovered to be a sign of immunological infiltration and a poor prognosis, as well as a potential and promising therapeutic target for tumors.

## 2. Results

The goal of this study is to investigate the relationship between ETS1 and immunology and to determine its predictive value as a possible biomarker in human cancers. We will look into the genetic abnormalities, expression patterns, and survival assessments of ETS1 expression in pancancer patients, as well as the relationship between ETS1 expression and tumor immune infiltration. Finally, we looked into the relationship between PPI and gene functional enrichment.

### 2.1. Clinical Landscape of ETS1 Expression

**Figure 1:**
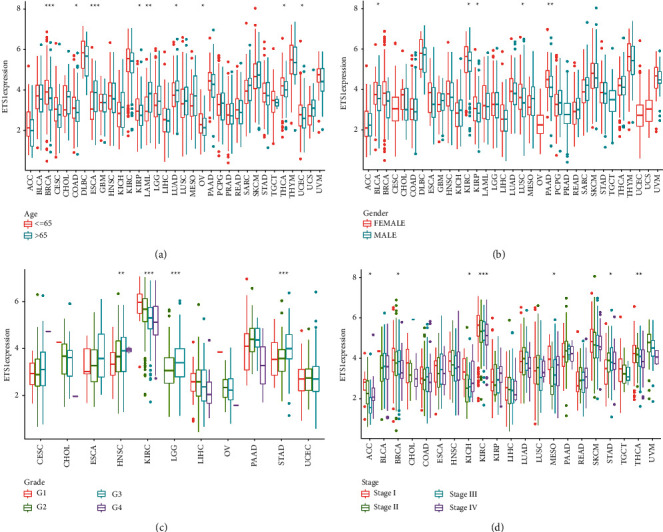
The clinical correlation of ETS1. (a) Age. (b) Gender. (c) Grade. (d) Stage.

ETS1 was expressed differentially in senior GBM patients, as indicated in Figure 1(a), but it was weakly expressed in ESCA, LAML, COAD, LUAD, UCEC, OV, KIRP, THCA, and BRCA. The findings revealed substantial gender differences in BLCA, KIRC, KIRP, LUSC, and PAAD expression (Figure 1(b)). Meanwhile, ETS1 expression is linked to grade stage in numerous cancers, including HNSC, KIRC, LGG, and STAD (Figure 1(c)). Furthermore, ETS1 expression is linked to tumor stage in a variety of malignancies, including BRCA, ACC, MESO, KICH, KIRC, STAD, and THCA (Figure 1(d)).

**Figure 2:**
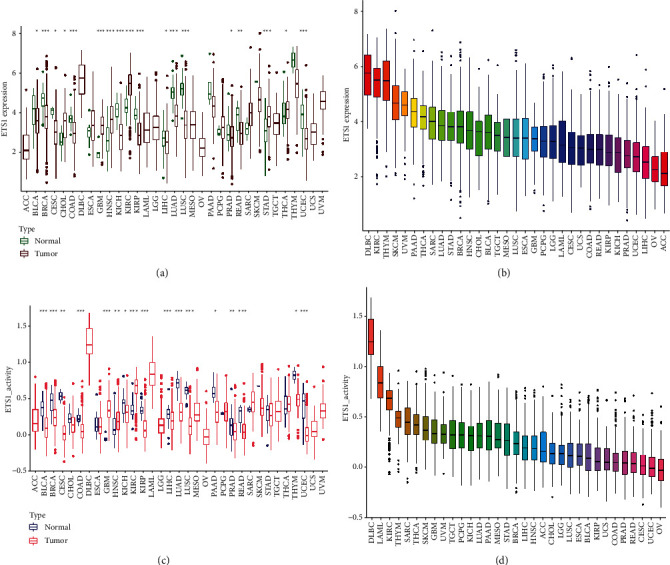
ETS1 activity. (a) Different analysis of ETS1. (b) The mean expression of ETS1. (c) Different activity analysis of ETS1. (d) The mean activity of ETS1.

ETS1 may be an essential new target or biomarker for cancer diagnosis since it can be a sensitive indicator. ETS1 mRNA expression was shown to be significantly higher in cancer samples from LUAD, LUSC, BLCA, READ, CESC, DLBC, KICH, KIRC, CHOL, GBM, HNSC, COAD, BRCA, PRAD, KIRP, LIHC, TGCT, THCA, and UCEC, indicating that ETS1 may act as an oncogene in the development of a variety of cancers (Figure 2(a)).  Figure 2(b) reveals that the expression levels of DLBC, KIRC, THYM, and SKCM are considerably more significant. As shown in Figure 2(c), ETS1 activity rose considerably in the tumor categories CHOL, ESCA, GBM, HNSC, KIRC, and KIRP, but was reduced in the tumor categories BLCA, BRCA, CESC, COAD, GBM, HNSC, KICH, KIRC, KIRP, LIHC, LUAD, LUSC, PAAD, PRAD, READ, THYM, and UCEC.  Figure 2(d) reveals that DLBC, LAML, KIRC, and THYM have much higher levels of activity.

### 2.2. ETS1's Prognostic Value in Cancer

**Figure 3:**
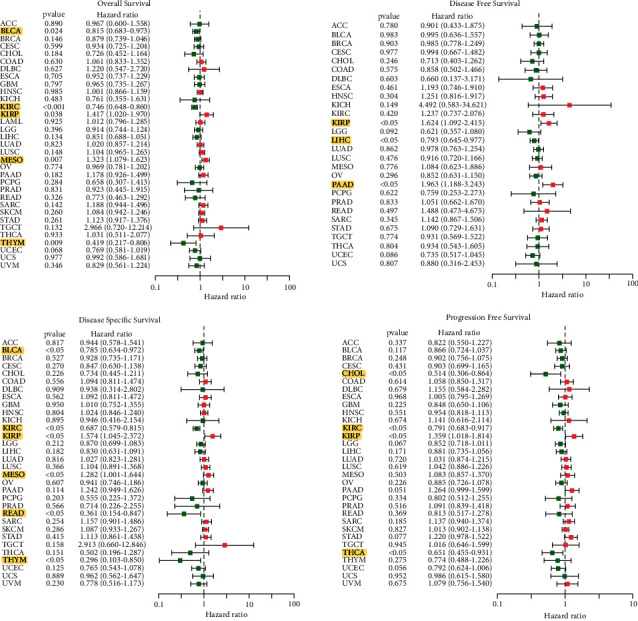
Univariate Cox regression analyses.

In KIRP and MESO, there was a positive association between ETS1 and OS, whereas in BLCA, KIRC, and THYM, there was a negative link. There was a clear positive connection between ETS1 and DFS in KIRP and PAAD. In KIRP and MESO, ETS1 expression is a risk factor, whereas it is a protective factor in BLCA, KIRC, READ, and THYM in DSS. The PFS further verified the protective effect of ETS1 in CHOL, KIRC, and THCA, as well as its significance as a risk factor in KIRP. The plot, on the other hand, allowed the researchers to discover other malignancies where ETS1 expression was thought to be a risk factor, such as KIRP and MESO. ETS1 expression was highly correlated with survival in many malignancies, although it is a fact that it was not directly connected to clinical characteristics (e.g., BLCA, KIRC, and THYM) (Figure 3).

### 2.3. ETS1 Expression and Immune Infiltrating Levels in *Cancer*

**Figure 4:**
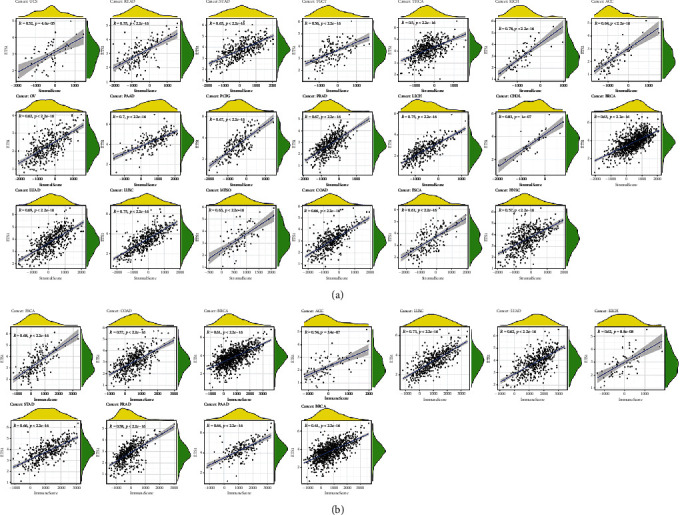
ETS1 expression and the ESTIMATE score. (a) StromalScore. (b) ImmuneScore.

**Figure 5:**
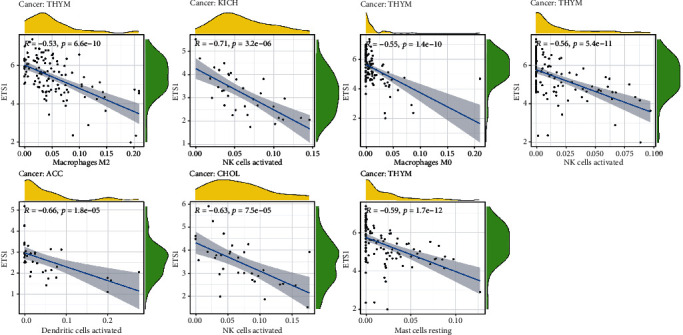
ETS1 expression and immune infiltration.

We also investigated whether ETS1 was related to the level of immune infiltration in diverse cancers. The stromal and immunological ratings are summarized in Figure 4. ETS1 expression has been linked to the stromal scores ACC, BRCA, CHOL, COAD, ESCA, HNSC, KICH, LIHC, LUAD, LUSC, MESO, OV, PAAD, PCPG, PRAD, READ, STAD, TGCT, THCA, and UCS, as well as the immune scores BRCA, ACC, CHOL, COAD, ESCA, KICH, LUAD, LUSC. ETS1 expression was related with dendritic cells activated in ACC, macrophages M0 in THYM, macrophages M2 in THYM, mast cells resting in THYM, and NK cells activated in THYM, CHOL, and KICH, as shown in Figure 5.

### 2.4. Analysis of ETS1 Expression and Immune Modulators

**Figure 6:**
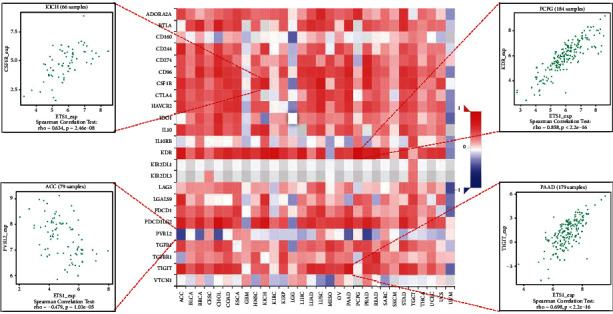
ETS1 and immune inhibitors.

**Figure 7:**
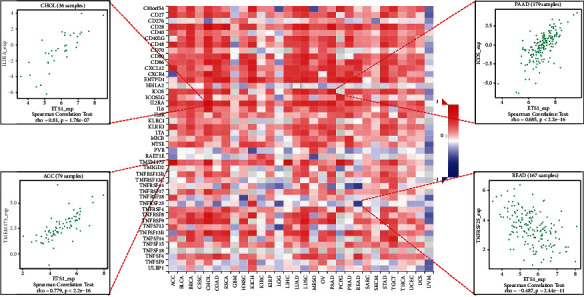
ETS1 and immune stimulators.

**Figure 8:**
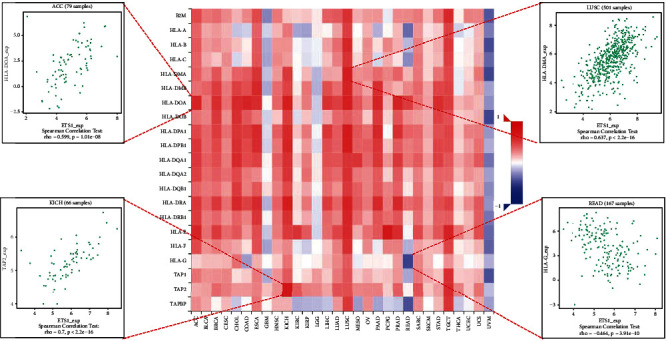
ETS1 and MHC molecules.

Figure 6 presents the investigation of 24 various types of immune inhibitors. ETS1 expression was positively related to CSF1R in KICH, KDR in PCPG, and TIGIT in PAAD but negatively connected with PVRL2 in ACC. Correlation studies of 45 immune stimulators (Figure 7) revealed that ETS1 expression was linked with IL2RA in CHOL, TMEM173 in ACC, and ICOS in PAAD but not with TNFRSF25 in READ. Furthermore, as shown in Figure 8, ETS1 expression was positively connected to HIA-DOA in ACC, TAP2 in KICH, and HIA-DMA in LUSC, but had a negative connection with HIA-G in READ.

### 2.5. Immunotherapeutic Markers and Response of ETS1

**Figure 9:**
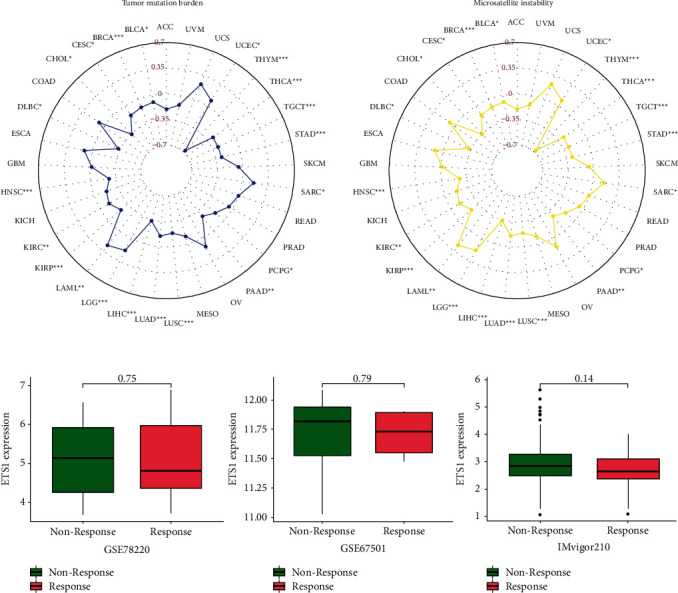
The relationship between ETS1 and immunotherapeutic indicators as well as response.

The connection with ETS1 and immune checkpoint blockage (TMB and MSI) was studied further.  Figure 9 shows that ETS1 is positively associated with TMB in CESC, BRCA, BLCA, UCEC, KIRC, LAML, LGG, LUAD, LUSC, PCPG, SARC, THCA, TGCT, and STAD but negatively related to CHOL, DLBC, HNSC, KIRP, LIHC, PAAD, and THYM. MSI was shown to have a positive association in BLCA, BRCA, CESC, KIRC, LAML, LGG, LUAD, LUSC, PCPG, SARC, TGCT, THCA, and UCEC and a negative association in CHOL, DLBC, HNSC, KIRP, LIHC, PAAD, STAD, and THYM.

Figure 9 shows that in the three cohorts, there is no statistically significant difference in ETS1 between responder and nonresponder groups. In the studied cohorts, patients with lower ETS1 levels were shown to be more susceptible to immunotherapy.

### 2.6. PPI Network of ETS1 in Cancers and GSEA

**Figure 10:**
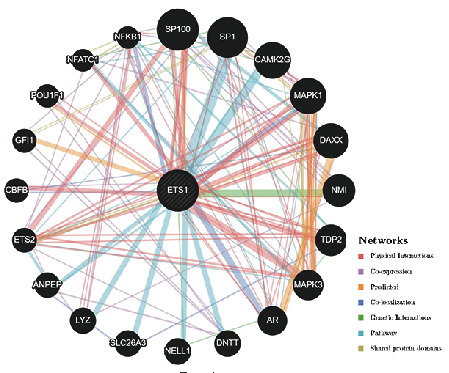
PPI network of ETS1.

**Figure 11:**
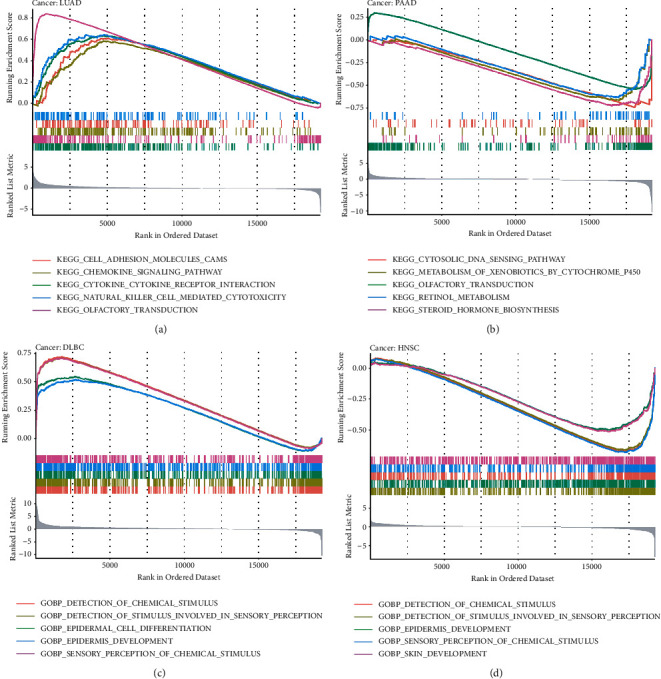
GSEA for samples with ETS1 expression. (a, c) The low expression. (b,d) The high expression sample. (a, b) KEGG. (c, d) GO.

Following that, we constructed an ETS1 PPI network to examine the underlying pathways by which ETS1 contributes to cancer carcinogenesis (Figure 10). ETS1 made solid physical contact with SP100, as seen in the image, which is essential for cancer spread. SP100 (SP100 nuclear antigen) is a protein-coding gene. ETS1 functions as a transcriptional coactivator and is involved in a variety of physiological processes including cell proliferation, differentiation, and death [12]. It may also act as an ETS1 corepressor, preventing it from binding to DNA in certain circumstances [13]. Regulation of ETS1 may have a role in angiogenesis by altering endothelial cell motility and invasion [14]. ETS1 was also expected to be related to SP1 and CAMK2G. The functional enrichment of high and low ETS1 expression was then determined using GSEA (Figure 11). High expression of ETS1 was mainly connected with metabolic-related activities such as cytosolic DNA sensing pathway, metabolism of xenobiotics by cytochrome p450, olfactory transduction, retinol metabolism, and steroid hormone biosynthesis, according to the KEGG enrichment term. According to the GO enrichment term, high expression of ETS1 is mainly linked with detection of chemical stimulus, detection of stimulus involved in sensory perception, *epidermis* development, sensory perception of chemical stimulus, and skin development.

## 3. Discussion

Inflammation is the body's principal defense mechanism. Many immunocytes and chemicals form a large regulatory network during inflammation, eliminating endogenous and external toxic substances to safeguard the organism. However, network imbalances, such as exaggerated inflammatory responses and a protracted inflammatory state, may exacerbate tissue damage [15, 16].

ETS1 is a toxicant-related transcription factor that plays an important part in the immunological TME and may have immunotherapeutic potential, contrary to popular belief. As a result, further ETS1-related research including TME, immune cells, immunological modulators, and the immunotherapeutic response is required. This research aimed to understand more about the pathways that may link ETS1 to immune-related factors in pancancer. First, the relationship between ETS1 and clinical factors was studied, and no significant changes in age, gender, or tumor stage were found in the majority of cancer types, supporting prior findings. ETS1 expression, on the other hand, has only marginal prognostic value in a variety of cancers, including gastric cancer (GC) [17]. Similarly, previous research has identified ETS1 as a proto-oncogene in various cancers, including hepatocellular carcinoma [18], colorectal cancer [19], and cervical *cancer* malignancy [20]. The RHPN1-AS1/miR-1299/ETS1 positive feedback loop accelerates GC degradation [21]. ETS1 promotes epithelial-to-mesenchymal transition and enhances transforming growth factor signaling in prostate cancer cells [22]. However, the role of ETS1 in TME invasion in diverse cancers remains unknown. Given ETS1's importance in the physiology of inflammation, it is a feasible possibility as a diagnostic biomarker and therapeutic target for inflammation-related diseases, and its clinical potential warrants further investigation.

Furthermore, when compared to the ETS1 activity score, the transcription level partially matched the total ETS1 activation in several tumors (e.g., BLCA, BRCA, CESC, COAD, GBM, HNSC, KICH, KIRC, KIRP, LIHC, LUAD, LUSC, PRAD, READ, and UCEC), indicating that the transcription level represented ETS1 activation in these tumors. ETS1 expression and activity were inconsistent in several cancers (PAAD, THYM, CHOL, DLBC, TGCT, and THCA). This might be due to ETS1 expression being influenced by posttranscriptional protein modification or protein metabolism.

To assess ETS1's potential utility, we further investigated the relationship between ETS1 and immune cell infiltration.

The link between ETS1 and immune cell infiltration was examined further to evaluate ETS1's potential use. ETS1 and M2 and M0 macrophages were discovered to have a significant connection in THYM. Moreover, some previous studies indicate that ETS1 impacts tumor growth and immune responses inside TME-associated macrophages [17]. ETS1 may be implicated in macrophage polarization and subsequent immunosuppressive response activation [23]. KDR would have the most significant adverse connection with ETS1 in PCPG. Except for UVM, most immune stimulants and MHC molecules showed a positive relationship with ETS1. This fascinating discovery might lead to the discovery of a unique regulatory mechanism in UVM immunotherapy. Furthermore, enrichment analysis revealed that high ETS1 expression was mostly connected with metabolic-related activities. Metabolic inflammation is defined by dysregulation of cytokine and adipocytokine expression in adipose tissue [24]. ETS1 is a transcriptional factor. In a variety of biological conditions, it directly affects the expression of cytokine and chemokine genes [25]. This protein may influence lymphoid cell development, survival, and proliferation and cause inflammatory molecules to clump together, making it easier for macrophages to enter [26]. According to the current findings, increased ETS1 expression may influence innate immunity in certain malignancies by activating metabolic-related pathways.

Furthermore, in this study, TMB and MSI were found to have a significant connection with ETS1 in various cancers. The TMB provides a decent estimate of tumor-neoantigen burden. The more somatic mutations a tumor has, the more probable it is to produce neoantigens [27]. On the other hand, MSI is described as a robust mutator phenotype caused by poor DNA mismatch repair and is a possible prognostic indicator for immunotherapy [28]. ETS1 was adversely associated with TMB and MSI in CHOL, DLBC, HNSC, KIRP, LIHC, PAAD, and THYM; however, it was positively associated with both biomarkers in BLCA, BRCA, CESC, KIRC, LAML, LGG, LUAD, LUSC, PCPG, SARC, TGCT, THCA, and UCEC. This suggested that ETS1 might have an indirect influence on the immunotherapeutic response to past cancers. The relationship between ETS1 and immunotherapeutic response was explored, but no statistically significant differences were discovered in any of the cohorts tested. As a result, our findings shed insight on ETS1's latent involvement in tumor immunology and its potential application as a cancer biomarker. Meanwhile, only three relevant cohorts were studied in our investigation of immunotherapeutic responses, making it difficult to define the precise immunotherapeutic response of ETS1. In the future, more important immunotherapeutic populations should be explored.

This study provided more information about the role of ETS1 in cancer immunotherapy. It reveals a relationship between ETS1 and critical immunological markers, which might help researchers better understand the potential linkages between ETS1 and the immune system. The present study has some limitations. The results provide a foundation for theoretical foundations and analytical concepts. We only built a verified ETS1 prediction signature by using the TCGA datasets and were unable to gather enough external data from other publicly available sources to verify the model's credibility. Furthermore, the bioinformatics research revealed some interesting details concerning ETS1's function in cancer. Biological research, both *in vitro* and *in vivo*, is required to confirm our results and improve treatment effectiveness.

## 4. Conclusions

In conclusion, our findings revealed a close relationship and prognostic significance of ETS1 expression in various human cancers. ETS1 can be a novel cancer treatment target. Our findings also provide insight into ETS1's important involvement in carcinogenesis and metastasis and a proposed mechanism through which ETS1 expression modulates tumor immunology and metabolic activity. Our findings can contribute to the identification of a relationship between ETS1 expression and immunological TME to further elucidate their possible function in cancer genesis and progression and thus provide immuno-based anticancer therapy.

## 5. Marerials and Methods

### 5.1. Acquisition and Processing of Raw Data

We obtained gene expression patterns and clinical information from The *Cancer* Genome Atlas (TCGA) [29] for 33 cancers. ACC (adrenocortical carcinoma); BLCA (bladder urothelial carcinoma); BRCA (breast invasive carcinoma); CESC (cervical squamous cell carcinoma and endocervical adenocarcinoma); CHOL (cholangiocarcinoma); COAD (colon adenocarcinoma); DLBC (lymphoid neoplasm diffuse large B-cell lymphoma); ESCA (esophageal carcinoma); GBM (glioblastoma multiforme); HNSC (head and neck squamous cell carcinoma); KICH (kidney chromophobe); KIRC (kidney renal clear cell carcinoma); KIRP (kidney renal papillary cell carcinoma); LAML (acute myeloid leukemia); LGG (brain lower grade glioma); LIHC (liver hepatocellular carcinoma); LUAD (lung adenocarcinoma); LUSC (lung squamous cell carcinoma); MESO (mesothelioma); OV (ovarian serous cystadenocarcinoma); PAAD (pancreatic adenocarcinoma); PCPG (pheochromocytoma and paraganglioma); PRAD (prostate adenocarcinoma); READ (rectum adenocarcinoma); SARC (sarcoma); SKCM (skin cutaneous melanoma); STAD (stomach adenocarcinoma); TGCT (testicular germ cell tumors); THCA (thyroid carcinoma); THYM (thymoma); UCEC (uterine corpus endometrial carcinoma); UCS (uterine carcinosarcoma); and UVM (uveal melanoma) were included in 33 types. The ETS1 status change was discovered using the cBioPortal database [30]. The genomic changes include copy number amplification, severe loss, an unknown missense mutation, and mRNA overexpression. The TCGA provides data on ETS1 expression differences between tumors and matched normal tissue. After extracting the ETS1 data with the Limma package, we used log2 (TPM+1) transformed expression data to illustrate the difference between the analysis findings in parameter selection.

### 5.2. The Relationship between ETS1 and Survival and Clinical Stage

Overall survival (OS), disease-specific survival (DSS), disease-free survival (DFS), and progression-free survival (PFS) were used to assess ETS1's influence on cancer survival. We employed the log-rank and univariate Cox proportional hazards models. Clinical factors such as age, gender, grade, and stage were considered for multivariate Cox regression. The stage survival plot module was used to evaluate the association between ETS1 expression and clinical stage.

### 5.3. The Role of Immune Cell Infiltration and the TME in ETS1 Expression

We investigated the link between ETS1 expression and tumor-infiltrating immune cell gene markers in malignant tumors and observed some immune cell infiltration. After assessing the TME with the ESTIMATE, the stromal score, immune score, and ESTIMATE scores were computed. Tumor purity was shown to be negatively related to the previously reported ratings. The Limma was then utilized to evaluate the variances in TME in several cancer samples according to the immunological, ESTIMATE, and stroma scores. To measure tumor cell purity, corresponding scatterplots were constructed.

Tumor mutation load (TMB) is a specific and accurate biomarker for predicting immunotherapy response. It can calculate the overall number of mutations per DNA megabase and identify alterations categorized as nucleotide insertions, base substitutions, or deletions [31]. MSI is a molecular tumor characteristic that is distinguished by the spontaneous loss or gain of nucleotides from short tandem repeat DNA sequences [32]. To study the link between TMB and MSI, we employed the fmsb package.

### 5.4. Immunotherapeutic Response Analysis

This study, as previously noted, included and assessed three major independent immunotherapeutic cohorts, namely, GSE78220, GSE67501, and IMvigor210. The respondents include patients who achieved a complete or partial response rather than nonrespondents who had either progressing disease or stable illness symptoms. The Wilcoxon test was then used to compare the levels of ETS1 expression in the respondent and nonrespondent groups.

### 5.5. Gene Set Enrichment Analysis and Network of Protein-Protein Interactions

Gene set enrichment analysis (GSEA) was carried out in both the high and low-expression groups. Based on the KEGG and GO analyses, the top four words were displayed. Enrichment was found to be significant in gene sets with |NES|>1, NOM *p* < 0.05, and FDR *q* < 0.05 [33]. In addition, we used the GeneMANIA web tool to create an ETS1 protein-protein interaction (PPI) network (https://www.genemania.org) [34].

## Data Availability

Patients who gave informed consent to use their data have been uploaded to publicly accessible TCGA databases. At their leisure, users can get and publish relevant articles depending on the needed data. The (diseases) data used to support the findings of this study have been deposited into the (TCGA) repository (https://portal.gdc.cancer.gov/).

## Abbreviations

ETS1: ETS proto-oncogene 1, transcription factor
GO: Gene ontology
KEGG: Kyoto Encyclopedia of Genes and Genomes
TME: Tumor microenvironment
TMB: Tumor mutational burden
DSS: Disease-specific survival
HR: Hazard ratios
PPI: Protein-protein interaction
CR: Complete response
PD: Progressing disease
TCGA: The *Cancer* Genome Atlas
GEO: Gene expression omnibus
GSEA: Gene set enrichment analysis
MSI: Microsatellite instability
OS: Overall survival
CI: Confidence intervals
SD: Stable illness
PFS: Progression-free survival
PR: Partial response.
